# Supplementary material for: Heparin-based hydrogel scaffolding alters the transcriptomic profile and increases the chemoresistance of MDA-MB-231 triple-negative breast cancer cells
Source: Biomater Sci. 2020 Feb 13;8(10):2786–96. doi: 10.1039/c9bm01481k (PMC7497406; doi:10.1039/c9bm01481k)
Supplement: Supplementary file 2 [file BM-008-C9BM01481K-s002.zip › Supplementary File 4/HP-BhydrogelvControl/Pathways/my_analysis.Gsea.1545201079907/HALLMARK_PANCREAS_BETA_CELLS.html]

Details for gene set HALLMARK\_PANCREAS\_BETA\_CELLS[GSEA]

|  || Dataset | expr.class.cls#HYD\_versus\_CONTROL.class.cls#HYD\_versus\_CONTROL\_repos |
| Phenotype | class.cls#HYD\_versus\_CONTROL\_repos |
| Upregulated in class | CONTROL |
| GeneSet | HALLMARK\_PANCREAS\_BETA\_CELLS |
| Enrichment Score (ES) | -0.5155117 |
| Normalized Enrichment Score (NES) | -1.5722667 |
| Nominal p-value | 0.03142857 |
| FDR q-value | 0.012242952 |
| FWER p-Value | 0.069 |
Table: GSEA Results Summary

  

Fig 1: Enrichment plot: HALLMARK\_PANCREAS\_BETA\_CELLS      
 Profile of the Running ES Score & Positions of GeneSet Members on the Rank Ordered List

  

| PROBE | DESCRIPTION (from dataset) | GENE SYMBOL | GENE\_TITLE | RANK IN GENE LIST | RANK METRIC SCORE | RUNNING ES | CORE ENRICHMENT || 1 | CHGA | na |  |  | 2238 | 1.261 | -0.0370 | No |
| 2 | VDR | na |  |  | 4898 | 0.641 | -0.1351 | No |
| 3 | MAFB | na |  |  | 5745 | 0.520 | -0.1464 | No |
| 4 | FOXO1 | na |  |  | 6270 | 0.447 | -0.1454 | No |
| 5 | PCSK1 | na |  |  | 6589 | 0.405 | -0.1365 | No |
| 6 | STXBP1 | na |  |  | 11982 | -0.170 | -0.4068 | No |
| 7 | DPP4 | na |  |  | 12540 | -0.229 | -0.4213 | No |
| 8 | FOXA2 | na |  |  | 12896 | -0.271 | -0.4227 | No |
| 9 | SYT13 | na |  |  | 13177 | -0.310 | -0.4178 | No |
| 10 | ELP4 | na |  |  | 14881 | -0.546 | -0.4720 | Yes |
| 11 | AKT3 | na |  |  | 15279 | -0.613 | -0.4540 | Yes |
| 12 | SRPRB | na |  |  | 15991 | -0.742 | -0.4442 | Yes |
| 13 | NKX6-1 | na |  |  | 17360 | -1.105 | -0.4457 | Yes |
| 14 | SEC11A | na |  |  | 17640 | -1.217 | -0.3833 | Yes |
| 15 | SRP9 | na |  |  | 17799 | -1.286 | -0.3103 | Yes |
| 16 | LMO2 | na |  |  | 18032 | -1.403 | -0.2337 | Yes |
| 17 | SPCS1 | na |  |  | 18745 | -1.947 | -0.1478 | Yes |
| 18 | SRP14 | na |  |  | 19097 | -2.712 | 0.0053 | Yes |
Table: GSEA details [plain text format]

  

Fig 2: HALLMARK\_PANCREAS\_BETA\_CELLS      
 Blue-Pink O' Gram in the Space of the Analyzed GeneSet

  

Fig 3: HALLMARK\_PANCREAS\_BETA\_CELLS: Random ES distribution      
 Gene set null distribution of ES for **HALLMARK\_PANCREAS\_BETA\_CELLS**
